# Supplementary material for: Are carbon emissions trading and green financial instruments synergistic? -Comprehensive quantitative research based on content analysis
Source: PLoS One. 2024 Mar 7;19(3):e0298601. doi: 10.1371/journal.pone.0298601 (PMC10919601; doi:10.1371/journal.pone.0298601)
Supplement: S1 Data — (ZIP) [file pone.0298601.s001.zip › Data/地级市Z_F工作报告.zip!/╜¡╦╒/╠⌐╓▌╩╨/└┤╫╘╣1⁄2╓┌║┼í╛┬φ┐╦╩2╛▌═°í┐_2015.docx]

各位代表：

　　现在，我代表市人民政府向大会作工作报告，请予审议，并请市政协委员和其他列席人员提出意见。

一、 2014年工作回顾

　　过去的一年，我们在中共泰州市委的正确领导下，坚持以党的十八大、十八届三中四中全会和习近平总书记系列重要讲话精神为指导，统筹做好稳增长促改革调结构惠民生各项工作，较好完成全年各项目标任务。预计实现地区生产总值3300亿元，增长10.6%。公共财政预算收入283亿元，增长9.2%。研发经费支出占地区生产总值比重2.3%。固定资产投资2200亿元，增长21.3%。社会消费品零售总额937亿元，增长12%。城乡居民人均可支配收入分别达31430元、15130元，分别增长9.5%、11.2%。居民消费价格涨幅2.1%。城镇登记失业率1.95%。

　　推进转型升级，强化创新驱动，经济发展提质增效。以实施“双百工程”为抓手，调整优化产业结构，推动产业向中高端迈进。工业经济稳中有进。规模以上工业实现增加值2170亿元，增长11.5%。深入实施新兴产业双倍增计划，实现产值3902亿元，增长16.8%。积极运用信息技术和先进适用技术改造提升传统产业，实施亿元以上重点工业项目279个，完成技改投入983亿元。装备制造业实现产值5215亿元，增长15%。完成省定化解落后产能年度任务。建筑业实现总产值2550亿元，增长15.2%。服务业发展不断加快。实现服务业增加值1390亿元，增长12%。新增省服务业创新示范企业3家，百亿级服务业集聚区达4家。大力发展旅游业，全年接待游客1800万人次，实现旅游总收入215亿元。加大金融服务实体经济力度，新增人民币贷款407亿元，净增直接融资117.5亿元。农业现代化建设成效明显。姜堰国家农业改革与建设试点绩效考评居全国首位，高港获批国家农业综合开发县，兴化建成国家级出口食品农产品质量安全示范区，海陵现代农业园区建成省级园区。科技创新能力进一步增强。高新技术产业产值突破4000亿元，增长17.8%，占规模以上工业比重达41.2%。加强自主品牌和标准体系建设，新创中国驰名商标7件，新获发明专利授权340件。强化人才支撑，新增省“双创计划”团队1个、人才15名，省“双创博士”25名。扎实抓好节能减排，单位地区生产总值能耗下降4%，主要污染物排放量削减完成年度目标任务。

　　深化综合改革，扩大对外开放，发展活力进一步释放。重点领域和关键环节改革不断深化。突出简政放权，出台并向社会公布行政事业性收费、行政权力、行政审批等目录清单，推行行政审批容缺受理、并联审批，率先实施工商登记负面清单制度，企业注册登记实行“三证合一”，4个镇（街）简政放权改革试点工作有序推进。市公共资源交易中心建成运行，排污权有偿使用和交易改革顺利实施。全面开展村级集体经济股份制改革，启动农村土地承包经营权抵押贷款试点。加强政府性债务和融资平台管理，严格执行“两个清单”、“两个审批”制度，落实偿债准备金制度。“营改增”试点企业达1.1万户，累计减税10.8亿元。开放型经济平稳发展。完善招商引资工作机制，制定项目大突破实施意见，加强驻点招商、小分队招商、产业定向招商，全年新批3000万美元以上项目24个，实际利用外资9.4亿美元。稳定外经外贸，完成进出口总额108.9亿美元，实现外经营业额7.9亿美元。加快中国医药城产业化步伐，签约落户医药类项目128个，在建产业化项目103个，新增投产企业30家。高港汽车零部件产业园创成省级科技产业园，锡山经济开发区兴化工业园、江阴高新区黄桥工业园和昆山高新区姜堰工业园先后获批。推进新一轮沿江开发，编制实施沿江转型升级规划，泰州港核心港区5万吨级集装箱码头开港运营。海关、国检、海事、边检为开放开发作出积极贡献。

　　提高组织程度，加快城乡建设，功能形象明显提升。中心城市建设取得新进展。坚持规划引领，组织开展第四轮城市总体规划修编，着力维护规划权威性和稳定性。调整优化市区城建体制，启动实施城建新提升两年行动计划。制定出台推进老城改造加快新城建设的意见，周山河新城开发建设全面提速，西客站片区、钟楼巷文化街区改造等项目加快实施。完善拆迁政策，盘活财政资金、国有资产、土地资源，积极化解拆迁和融资难题。加强城市管理标准化建设，加大违法建设防控力度，市容市貌持续改善。城乡基础设施建设取得新突破。泰镇高速泰州段、阜兴泰高速兴化至泰州段、京沪高速江广段扩容开工建设，宁启铁路复线电气化改造泰州段主体工程基本完工，国电泰州电厂二期、连淮扬镇与宁启铁路东北联络线获批，泰州常州、江阴靖江过江通道列入国家规划。市区东风路南段、永定路东段快速化改造和环城东路东环高架建设有力推进，梅兰路、育才路等断头路相继接通。泰东河整治、引江河二期、中小河流治理等工程进展有序。办好新一轮农村实事，区域供水通水乡镇、污水处理设施实现建制镇全覆盖，农村饮水安全工程新增受益人口83万人，新改建农村道路295公里、桥梁167座，疏浚县乡村河道995条。生态文明建设取得新成效。市本级通过省级生态市考核验收，姜堰、海陵分别通过国家级生态区考核验收和技术评估，新增33个国家级生态乡镇。落实生态红线保护规划，加强生态环境保护修复。城市环境综合整治扎实推进，村庄环境整治通过省级全域考核验收，并获省人居环境范例奖。加强绿化建设，实施“空转绿”工程，全市新增造林面积7万亩，市区新增绿地面积100万平方米。

　　优化公共服务，增进民生福祉，社会保持安定和谐。大力实施民生幸福工程，认真办好改善民生实事。鼓励支持全民创业，新增创业6.8万人，净增私营个体经济注册资本668亿元。实施更加积极的就业政策，突出抓好重点人群就业，新增城镇就业10万人。推进社会保障扩面提标，社会保险主要险种覆盖率稳定在97%以上，城镇居民大病医疗保险实现全覆盖，城乡居民和企业离退休人员养老金、新农合和城镇居民医保筹资、城乡低保标准进一步提高。加强社会救助工作，发展慈善福利事业，市残疾人就业服务中心获“全国残疾人之家”称号。全市新开工各类保障性住房1.6万套，住房公积金新增开户缴存职工4.7万人。扶持发展养老服务业，新改建养老机构32家，新增养老床位5236张。实施脱贫奔小康工程，新增3.3万农村低收入人口脱贫。努力办好人民满意教育，学前教育省优质园比例达61%，各市（区）全部建成国家义务教育发展基本均衡市（区），“泰微课”自主学习平台上线运行，职业教育国际合作迈出新步伐，泰州学院新校区正式启用。深化医疗卫生资源整合，完善医疗卫生服务功能，基层医疗卫生机构建设步伐加快，县级公立医院改革全面实施，各市（区）全部建成国家中医药先进单位。积极发展文化事业和产业，市级公共文化设施全部免费开放，靖江创成省公共文化服务体系建设示范区；泰州文化创意产业园、黄桥乐器文化产业园获批省级文化产业示范园区，新增3家省重点文化科技企业。全国文明城市创建取得重要进展，各市（区）全部通过国家卫生城市技术评估。加强人口计生工作，稳步实施“单独两孩”生育政策。建成城市社区“10分钟体育健身圈”，启动市区学校室外体育设施向社会开放试点。新辟优化市区公交线路11条，公共自行车系统一期建成投用。深化平安泰州、法治泰州建设，群众安全感连续多年位居全省前列，我市被评为全国“六五”普法中期先进城市。全面落实社会稳定风险评估制度，加强和创新调解组织建设，有效预防和化解社会矛盾。健全立体化现代治安防控体系，严厉打击各类违法犯罪活动。应急管理体系不断完善，安全生产形势总体稳定，食品药品安全工作得到加强。国防教育和双拥共建深入开展，退役士兵村官培养工作成效突出。社会科学、广播电视、民族宗教、外事侨务、妇女儿童和气象、防震、人防、无线电管理等工作取得新进步。

　　转变工作作风，提升行政效能，履职能力不断增强。坚持把软环境建设作为硬任务来抓，进一步提高办事效率、优化政务服务。认真开展党的群众路线教育实践活动，贯彻执行中央八项规定和省、市委十项规定，扎实抓好“四风”问题整改落实。深入开展“三服务”活动，定期组织企业家座谈，进一步强化扶持政策落实和要素协调保障，切实为企业解难题、办实事。坚持民生工作例会制度，用好12345·政风行风热线、市长信箱和望海楼论坛三个平台，及时梳理解决群众反映强烈的突出问题。推进依法行政，加强法治政府建设，自觉接受人大的法律监督、工作监督和政协的民主监督，主动接受社会监督，强化行政监察和审计监督，广泛听取各民主党派、工商联、无党派人士和社会各界意见。认真办理人大代表建议和政协提案，落实率明显提高。积极打造“阳光政务”，健全政府信息发布制度，官方微博、微信影响力不断提升。加强惩防体系建设绩效评估，廉政风险防控机制加快完善。严肃查处腐败案件，着力解决发生在群众身边的不正之风和腐败问题。大力整治“庸懒散”现象，加强对重大项目、重点工作的督查推进，机关执行力进一步提升。

　　过去一年的成绩来之不易，这是中共泰州市委正确领导的结果，是全市上下攻坚克难、团结奋斗的结果。我代表市人民政府，向全市广大干部群众，向全体人大代表、政协委员，向各民主党派、工商联、无党派人士、各人民团体和广大离退休老同志，向中央和省驻泰机构以及驻泰部队指战员、武警官兵和政法干警，向所有关心、支持泰州发展的海内外朋友，表示衷心的感谢！

　　我们也清醒地看到，经济社会发展中还存在一些问题和不足，主要是：经济下行压力加大，有效投入相对不足，资源环境约束加剧，做大经济总量与提升发展质量形成双重挑战；全面深化改革进展还不快、亮点还不多，改革红利还没有得到充分释放；社会矛盾复杂多变，维护社会和谐稳定面临较大压力；政府职能转变还不到位，依法行政能力亟需提高，机关作风和效能仍需进一步改进，等等。这些问题，我们一定高度重视，积极应对，妥善解决。

二、 2015年工作任务

　　今年是全面完成“十二五”规划的收官之年、全面深化改革的关键之年、全面推进依法治国的开局之年，也是市委三大主题工作的全面启动之年。政府工作的总体要求是：深入学习贯彻党的十八大、十八届三中四中全会和习近平总书记系列重要讲话精神，认真落实中央经济工作会议、省委十二届九次全会、市委四届八次全会决策部署，主动适应经济发展新常态，以转型升级为主线，以全面深化改革为动力，以创新驱动发展为核心，以增进民生幸福为根本，坚持稳中求进，致力稳中奋进，大力推进“思想再解放、项目大突破、城建新提升”三大主题工作，促进经济平稳健康发展和社会和谐稳定。

　　全市国民经济和社会发展主要预期目标为：地区生产总值增长10%左右，公共财政预算收入、城乡居民收入实现与经济基本同步增长，固定资产投资增长20%以上，社会消费品零售总额增长12%左右，居民消费价格涨幅3%左右，城镇登记失业率控制在3%以内，研发经费支出占地区生产总值比重2.35%以上，节能减排完成省定目标任务。

　　面对复杂多变的宏观经济环境和艰巨繁重的改革发展任务，做好今年工作必须全面认识新常态下的新趋势新特征，自觉遵循新常态下的新规律新要求，牢牢把握经济社会发展主动权。注重转变工作方式。顺应转变经济发展方式的要求，加快转变政府工作理念和工作方式。更加自觉维护公开公正的市场环境，促进市场在资源配置中起决定性作用，保障各类企业公平参与市场竞争。更加尊重企业的市场主体地位，减少直接干预企业微观经济活动，真正发挥好政府的推动和引导作用。更加主动为企业提供高效周到的服务，帮助解决企业发展中的困难和矛盾。注重科学理性发展。系统谋划发展格局，挖掘资源禀赋，放大比较优势，着力在港口建设、互联网经济发展、生物医药产业前延后伸等方面发现培育新的经济增长点。与时俱进推进工作，紧扣年度目标任务，急而不躁抓发展、实而不浮搞建设，准确把握“好”与“快”的平衡点，力求在“好”的前提下能快则快。未雨绸缪防控风险，积极防范政府性债务、金融领域等潜在风险，牢牢守住不发生区域性系统性风险的底线。注重提高质量效益。深化产业结构调整，大力发展先进制造业，加快建立现代产业体系。发挥创新驱动作用，深入推进科技创新、制度创新、管理创新和商业模式创新，促进经济发展向“智慧型增长”跃升。提高集约发展水平，积极培育金融、技术、人才、土地市场，大力发展绿色低碳循环经济，进一步增强资源环境承载力。注重体现民生为本。找准民生改善与经济发展的结合点发力点，促进经济建设与民生建设有效互动、良性循环，实现“做大蛋糕”与“分好蛋糕”同步推进。落实民生投入长效机制，调整优化财政支出结构，确保民生投入足额到位、逐年增加。坚持积极作为、量力而行，注重民生工作的稳定性、连续性、积累性，立足当前办实事，着眼根本建体系，让群众真正得益受惠。

　　今年将重点抓好八个方面工作：

　　1．调优结构促转型，保持经济平稳健康发展。把转方式调结构放到更加重要位置，突出创新驱动，促进两业融合，提高经济发展的质量和效益。

　　加快中国医药城产业化进程。继续举全市之力建设中国医药城，推动医药城由药品研发生产向医疗服务和健康产业延伸，努力打造大健康产业基地。推进疫苗、诊断试剂及高端医疗器械等产业项目建设，新增药品投产企业12家，新落户医药企业100家以上。加快康健医疗区、标准厂房区五期等产业载体建设，组建生物医药产业母基金。推动部省共建不断深化，积极申报国家药品进口口岸城市，力争获批国家疫苗临床评价中心、新药创制重大专项产业化基地。

　　提升先进制造业发展水平。培育壮大新兴产业，引导企业更加注重研发能力和综合劳动生产率的提高，更加注重产品市场前景和销售服务网络的建立，力争实现新兴产业产值4600亿元。改造提升传统产业，支持企业加快生产工艺革新和技术装备现代化，促进信息化与工业化深度融合，推动生产制造向智能化、网络化方向发展。扶持企业做优做强，大力发展民营经济，力争净增规模以上企业150家以上，主营业务收入超50亿元企业达30家，新增主板上市企业1-2家。鼓励企业“走出去”，落实稳定外贸增长的政策措施。推进建筑工业化，拓展建筑业发展空间。

　　大力发展服务业。围绕现代物流、金融保险、软件和信息服务、工业设计等重点，加速发展生产性服务业。推进现代服务业集聚区扩容提质，新增市级以上现代服务业集聚区3-5家。制订实施旅游业发展三年行动计划，科学安排重大项目的布局、开发和建设，促进旅游业与健康产业、文化产业、城乡建设融合发展。加快城市重点商贸项目和社区商业设施建设，完善城乡商贸流通服务体系。积极培育新的消费市场和消费热点。

　　激发科技创新潜力。强化“四个对接”，着力形成有利于出创新成果、有利于创新成果产业化的新机制。认真落实“人才强企”十条新政策，全力打造人才高地，助推企业转型升级。探索推行“科技创新券”等激励措施，引导企业加大科技投入。推动产学研深度融合，加快建设泰州科技人才广场、产业研究院，力争新建市级以上工程技术研究中心30家。抓好科技与金融结合试点，加强科技支行、科技担保、科技小额贷款公司等专营机构建设。

　　2．聚焦项目大突破，增强经济发展的支撑和后劲。把项目建设作为经济工作的第一抓手，以增量优化带动存量调整，为转型升级、跨越发展夯实基础。

　　强势推进招商引资。把握招商重点，突出招引具有产业性质的重大基础设施类、教育研发培训类战略型项目，招引投资强度大、产出规模大、税收和就业贡献大的龙头型项目，招引有利于提高产业集中度、关联度和承载力的配套型项目。认真落实年度招商计划，精心组织北上广深投资促进周、泰台经贸交流合作促进月等活动；注重将招商引资的功夫下在平时，扎实做好项目信息收集、甄别研判和跟踪洽谈，进一步提高招商实效。强化招商队伍建设，培养一批高素质的专业招商人员。

　　加强开发园区建设。按照适度超前、科学定位的原则，力争完成省级以上开发区及特色园区规划修编。推进重点园区提档升级，支持出口加工区创建国家级综合保税区，泰兴经济开发区、高港高新区创建国家级经济开发区，兴化经济开发区创建国家级食品工业园区。加强多层标准厂房建设，加大闲置土地清理力度，促进开发园区集约化发展。鼓励发展“飞地经济”，推动实现良性竞争、合作共赢。优化开发园区管理体制，激发园区活力，加快转型升级。

　　落实项目建设机制。严格执行重大项目绿色通道制度，进一步优化审批流程，促进项目建设提速提效。严格执行领导挂钩联系制度，及时协调解决项目实施过程中的矛盾问题。严格执行“五个一”督查推进制度，严把项目签约落地、开工建设、竣工达产时间节点，分阶段过堂督查项目招引建设情况。严格执行滚动式项目管理和绩效考核办法，强化对投资强度、税收贡献、社会就业、节能环保等方面的综合考评。全年力争产业投资突破2000亿元，新开工过亿元产业项目180个以上。

　　3．突出城建新提升，提高中心城市首位度。强化规划引领，加快重点工程建设，进一步优化城市结构，完善城市功能，提升城市品质。

　　推进老城改造。深化历史文化名城保护，落实城中、渔行历史街区整治方案，建成评书评话博物馆、南山寺景区，完成稻河古街区改造复兴工程。加快西客站片区“一站一路一广场”建设，实施老旧小区、城中村、棚户区、后街背巷综合整治，推进钟楼巷、纺织新村等片区改造。加快西大门、北大门和西北片区环境综合整治，推进济川路、海陵路、税东街等路段立面出新。实施空转绿“311”工程，抓好西南城河景观绿化建设。加强社会停车场、农贸市场、公共厕所、垃圾收集处理等设施建设，推进西北工业园区、农业开发区污水管网全覆盖。

　　加快新城建设。进一步拉开道路框架，年内形成周山河以北的新城路网。突出抓好“1+2+3+N”教育布局工程建设，加快新区人民医院、中心血站和120急救中心、体育公园一期等项目建设，推进金融服务区和医药城会展中心二期建设。构建现代城市绿地系统，加大主题公园、生态廊道建设力度，启动建设郊野公园，实施周山河—天德湖景观提升工程。加强河湖水系保护、连通和管理，打造周山河、南官河、凤凰河生态休闲风光带。

　　优化完善交通。大力推进泰镇高速泰州段、阜兴泰高速兴化至泰州段、京沪高速江广段扩容建设，加快实施东风路南段和永定路东段快速化改造，积极做好盐泰锡宜、沪泰宁城际铁路前期工作。推进扬泰机场一类航空口岸对外开放，力争年内开通国际航线。深化泰州港发展战略研究，推动港口开发建设。加快鼓楼路北延、站前路一期、328国道和姜高路东延等重点道路建设，确保海阳路、吴陵路、引凤路等一批断头路接通。大力发展公共交通，进一步优化线路设置，提高公交出行分担率。推进高港汽车南站及公交枢纽建设，完成公共自行车系统二期工程。

　　4．深化改革激活力，着力构建体制机制新优势。务实推进既具有年度特点又有利于长远制度安排的改革举措，有效激发市场活力和社会创造力。

　　推进行政管理体制改革。抓好政府职能转变和机构改革工作，调整优化组织结构。加大简政放权力度，建立行政审批事项动态清理机制，深化市区管理体制改革，推进经济发达镇、重点镇（园区）简政放权改革试点。开展医药高新区行政审批制度改革试点，实现“园内事园内结”。强化事中事后监管，健全守信激励和失信惩戒机制。推动行业协会商会与行政机关脱钩，规范中介机构服务行为。有序实施公车改革。

　　推进财税体制改革。实施全口径预算管理，编制三年滚动财政规划，提高财政预算统筹能力。加大财政专项资金绩效评价和整合力度，推行拨改贷、拨改投、拨改股等产业扶持方式。深化税收征管改革和综合治税平台建设，完善税收征管质量监管机制。组建市国有资产经营（集团）公司，提高国有资产规模化经营水平。规范政府性债务管理，完善动态监控、风险预警机制。

　　推进资源要素市场化配置改革。建立新增工业用地弹性出让制度，全面开展农村土地承包经营权确权登记颁证，积极开展宅基地使用权流转试点。鼓励民间资本投资入股金融机构、发起设立中小型民营银行，吸引境内外私募股权机构在泰建立投资基金和分支机构。推进投融资体制改革，探索政府与社会资本合作等多种投融资模式。推行差别化排污总量控制和排污权交易制度，出台生态补偿和监管考核办法。

　　推进社会事业领域改革。深化考试招生制度改革，完善义务教育阶段学校就近免试入学制度。推进公立医院管理体制和人事管理、收入分配制度改革，健全医疗卫生机构运行机制。吸引民间资本、社会力量参与文化事业建设、产业发展，加快公共文化服务多元化社会化步伐。实施机关事业单位养老保险制度改革。

　　5．践行法治新要求，进一步提升依法行政水平。深入贯彻依法治国基本方略，切实加强法治政府建设，更好地运用法治思维和法治方式行使权力、履行职责。

　　提高行政决策水平。坚持依法科学民主决策，严格落实“公众参与、专家论证、风险评估、合法性审查、集体讨论决定”重大行政决策法定程序，确保决策制度科学、程序正当、过程公开、责任明确。推行政府法律顾问制度，调整充实法律专家咨询论证委员会。完善决策实施后评估制度，建立终身责任追究制度和责任倒查机制。加强对行政决策落实情况的监督，确保决策执行有力、落到实处。

　　推进公正文明执法。按照减少层次、整合队伍、提高效率的原则，合理配置执法力量。完善行政执法裁量权基准制度，探索设立综合执法协调机构，加大关系群众切身利益的重点领域行政执法力度。落实行政执法责任制，建立执法全过程记录制度，健全纠错问责机制。推进行政执法与刑事司法有效衔接，防止随意执法、以罚代刑。改革行政复议体制，加强相对集中行政复议权工作。

　　规范行政权力运行。推进行政机构、职能、权限、程序、责任法定化，做到法定职责必须为、法无授权不可为。加强对政府内部权力的制约，对权力集中的部门和岗位实行分事行权、分岗设权、分级授权，防止权力滥用。自觉接受人大法律监督、工作监督和政协民主监督，重视社会监督、舆论监督；深化行政监察和审计监督，对公共资金、国有资产、国有资源和领导干部履行经济责任等情况实行审计全覆盖。

　　6．推进新型城镇化，促进城乡发展一体化。坚持以人的城镇化为核心，以小城市建设为重点，夯实现代农业根基，守住生态文明底线，推动城乡统筹协调发展。

　　提高城镇化质量。加快完善新型城镇化和城乡发展一体化相关规划，推动城镇建设、土地利用、产业发展等“多规融合”。把小城市作为新型城镇化的关键节点，高标准推进基础设施和配套功能建设，促进差别化、特色化发展。坚持以产业化带动城镇化，增强城镇吸纳就业能力。推动农业转移人口市民化，提高户籍人口城镇化率，落实流动人口“同城待遇”。优化镇村公共服务体系，积极推进城乡低保、基本养老、医疗保险制度全面并轨。启动实施黄桥老区富民强村行动，逐步解决里下河地区饮用长江水问题。

　　发展现代农业。积极推进国家现代农业示范区建设，大力发展高效农业、生态农业，打造城市北部生态农业走廊。加快转变农业发展方式，培育新型农业经营主体和现代职业农民，构建新型农业经营体系，提高土地产出率、资源利用率和劳动生产率。加强农产品质量安全监管，推行农业标准化生产。完善强农惠农政策，改进农业补贴办法。扎实推进农田水利基础设施建设，加快实施川东港整治、马甸水利枢纽等重点水利工程。着力抓好靖江中农办农村改革试验联系点、省农村改革试验区和姜堰国家农业改革与建设试点工作。

　　改善生态环境。扎实开展生态系列创建，确保靖江、泰兴、海陵、高港、姜堰建成国家级生态市（区），兴化通过国家级生态市考核程序，力争省级经济开发区全部建成生态工业园区。深入实施主体功能分区战略，着力构建科学合理的国土空间开发与保护格局。突出抓好大气污染防治，认真落实扬尘治理、秸秆禁烧等关键举措，加快淘汰高污染燃料锅炉。推进全国水生态文明城市建设试点，完成城市黑臭河道整治任务。深化城市环境综合整治，建立村庄环境整治长效管理机制。

　　7．心系群众惠民生，全力维护社会和谐稳定。以实现共同富裕、共享发展成果为导向，深化拓展民生幸福工程，统筹发展民生社会事业，努力让全市人民过上更好生活。

　　力促城乡居民增收。大力扶持全民创业，让更多的人通过创业增加经营性收入，全年新增创业5万人，带动就业6万人以上。规范发展劳动力中介机构和市场，以更有针对性的就业服务解决劳动用工的结构性矛盾，再造泰州劳动力新优势。严格落实企业最低工资制度和工资集体协商、支付保障制度，稳步提高城镇职工工资性收入。落实“十项富民行动计划”，拓宽农民持续增收渠道。进一步完善最低生活保障和重点优抚对象、被征地农民社会保障制度，不断提高保障标准。全面推行定位扶贫、精准扶贫，统筹抓好经济薄弱村集体经济发展、低收入农户增收致富工作。加强保障性住房建设和管理，确保质量安全和公平分配。创新社会养老服务模式，支持发展慈善公益事业。

　　大力发展社会事业。以教育现代化建设为统领，促进教育公平，提高教育质量。扩大学前教育优质资源覆盖面，推进义务教育现代化学校标准化建设和素质教育“5+2”工程，进一步巩固基础教育优势。加快构建现代职业教育体系，强化高等教育统筹规划和资源整合。积极推进健康城市建设，稳步提升基本公共卫生服务水平。支持市人民医院、中医院做优做强，加快市妇幼保健院等公益性医疗机构建设。加强计生管理和服务，协调发展群众体育和竞技体育、体育事业和体育产业。

　　切实加强文化建设。倡导和弘扬社会主义核心价值观，培育形成奋发有为、崇德向善的良好风尚。加快建设现代公共文化服务体系，繁荣发展社会科学、广播影视、新闻出版、文学艺术事业。坚持项目带动、园区集聚和产业富民导向，推动文化产业跨界融合发展，打造富有特色的吉祥文化产业。着力挖掘优秀传统文化，不断深化“康泰之州、富泰之州、祥泰之州”城市主题文化宣传。巩固全国文明城市创建成果，健全文明城市创建长效机制。推进全民阅读，加强科学普及。

　　积极创新社会治理。强化基层基础工作，推动社会治理重心下移，健全社区管理体制和服务网络。深入开展领导干部接访下访，全面推行“阳光信访”和信访法治化。完善立体化现代治安防控、矛盾纠纷预防化解、人口服务管理等工作体系，进一步提高群众安全感。加强公共法律服务，扩大法律援助覆盖面。严格落实安全生产责任，杜绝重特大安全事故发生。强化食品药品安全专项整治。健全应急管理体制机制，增强灾害防御能力。深化双拥共建和国防教育，提高转退军人安置和优抚工作水平。

　　8．勤政廉政树形象，切实加强政府自身建设。适应新常态、谋求新作为，必须更加注重转变职能、创新管理、廉洁从政，不断增强政府公信力和执行力。

　　加快建设学习型政府。推进学习型班子、学习型机关建设，引导全体公务人员争当勤奋学习、善于思考的模范，解放思想、与时俱进的模范，勇于实践、锐意创新的模范。完善市政府中心组学习制度，落实领导干部学习调研联系点制度，准确把握宏观形势、政策导向和基层动态，不断提高引领发展新常态的能力和水平。坚持把推进思想再解放贯穿于政府工作全过程，注重运用改革创新的办法解决发展难题，精心组织“十三五”规划编制，进一步增强政府工作的预见性、主动性、实效性。

　　加快建设服务型政府。把为基层服务、为群众服务、为投资者服务作为应尽的职责，着力营造“新三比”服务环境。加强政府公共服务绩效管理，扩大政府购买服务范围，努力提供更多更好的公共产品。推进政务服务中心和公共资源交易中心标准化规范化制度化建设，不断改进政务服务方式。认真执行直接联系服务群众十项制度，切实办好12345·政风行风热线、市长信箱和望海楼论坛。深入推进政务公开，重点做好政府和部门预决算、“三公”经费公开工作。

　　加快建设廉洁型政府。巩固扩大党的群众路线教育实践活动成果，坚持不懈纠正“四风”，严肃整治懒政庸政。大兴艰苦奋斗、勤俭节约之风，牢固树立过紧日子的思想，把有限财力用在发展经济和改善民生上。坚持用制度管权管事管人，建立决策科学、执行坚决、监督有力的权力运行体系，从源头上预防和减少腐败现象发生。落实党风廉政建设责任制，加大惩治腐败力度，严肃查处发生在重点领域、关键环节和群众身边的腐败案件。推进廉政文化建设，加强反腐倡廉和警示教育，让全体公务人员受警醒、明底线、知敬畏，树立为民务实清廉形象。

　　各位代表，新常态新形势催人奋进，新目标新征程任重道远。让我们紧密团结在以习近平同志为总书记的党中央周围，在省委、省政府和市委的坚强领导下，团结拼搏，攻坚克难，扎实工作，为谱写“迈上新台阶、建设新江苏”的泰州篇章而努力奋斗！
